# The Relationship Between Self-Control and Non-Suicidal Self-Injury in Adolescent Psychiatric Outpatients: Exploring the Role of Self-Control

**DOI:** 10.3390/children12010099

**Published:** 2025-01-16

**Authors:** Zhenhua Chen, Jie Xu, Ronghua Zhang, Yuxuan Wang, Ziwei Shang

**Affiliations:** 1 Mental Health Center, Renmin Hospital of Wuhan University, Wuhan 430064, China; chenzh201308@163.com; 2 Institute of Developmental and Educational Psychology, School of Marxism, Wuhan University, Wuhan 430072, China; xujie@whu.edu.cn (J.X.); yuxuanwang@whu.edu.cn (Y.W.); shangziwei@whu.edu.cn (Z.S.)

**Keywords:** non-suicidal self-injury (NSSI), self-control, adolescents

## Abstract

Background: Non-suicidal self-injury (NSSI) is a significant public health concern that threatens the physical and mental health of adolescents. Given its high prevalence among adolescents, understanding the characteristics and contributing factors of NSSI is crucial. This study aimed to characterize NSSI and examine the relationship between self-control and NSSI among adolescent psychiatric outpatients. Method: This study was conducted in a psychiatric department of a hospital in Hubei Province, China, involving 206 adolescent psychiatric outpatients (135 females, 12–18 years old). Assessments included the Ottawa Self-Injury Inventory (OSI), the Self-Control Scale (SCS), and a self-designed sociodemographic questionnaire. Result: In this sample, 77.18% reported a history of NSSI. The prevalence of NSSI was significantly higher in females than in males (χ^2^ = 19.059, *p* < 0.01). The NSSI group had significantly lower self-control scores compared to the non-NSSI group (F = 27.458, *p* < 0.01). In the NSSI group (*n* = 156), self-control was negatively associated with NSSI frequency and fully mediated by NSSI function. Conclusions: These findings highlight the complete mediating role of NSSI function between self-control and NSSI frequency, offering insights for future prevention and intervention efforts.

## 1. Introduction

Non-suicidal self-injury (NSSI) is prevalent among adolescent populations and represents a significant public health issue, which warrants immediate attention [1,2]. NSSI is typically defined as a series of repeated, intentional, direct harm to the body, and it is generally not accepted and recognized by society [3]. NSSI is most common in adolescence, peaking in mid-adolescence, and decreasing with age. A 15-year longitudinal study of adolescent self-harm (*n* = 1943) found that adolescent self-harm behaviors typically occur between ages 14 and 19, peak around age 15, and decrease in frequency with age in late adolescence and early adulthood [4].

In non-clinical samples, the lifetime prevalence of NSSI among adolescents ranges from 13% to 45% [5]. In adolescent psychiatric samples, this rate is much higher, reaching 40–60% among adolescents and 19–25% among adults [6]. Repeated NSSI not only results in direct and potentially irreversible physical harm but also negatively impacts mental health, personality development, and increases the risk of suicidal behavior [7]. In a case series study on adolescents with a recent history of NSSI, 87.6% met the criteria for at least one DSM-IV diagnosis, including major depressive disorder (41.6%), post-traumatic stress disorder (23.6%), and generalized anxiety disorder (15.7%) [8]. Psychological symptoms such as depression and anxiety have been identified as potential risk factors for NSSI among adolescents [9,10].

### 1.1. The NSSI Function and NSSI Frequency

Why do adolescents commit NSSI and what is the purpose of doing so? The function of NSSI refers to the reason or purpose behind the behavior, which has been discussed for decades. According to Nock’s comprehensive theoretical model, NSSI is not only a way to regulate one’s emotional/cognitive experience, but also a way to communicate with or influence others. The main functions of NSSI can be categorized along two key dimensions: automatic versus social reinforcement and positive versus negative reinforcement. Specifically, on the automatic side, NSSI may serve as a form of automatic negative reinforcement, helping individuals avoid unpleasant emotional states, or as automatic positive reinforcement, allowing them to achieve a desired emotional state. On the social side, NSSI can function as social positive reinforcement by encouraging help-seeking or attention from others, or as social negative reinforcement by providing a way to escape unwanted social situations [11]. In 2007, Klonsky reviewed 18 studies and summarized seven primary functions of NSSI: affect regulation, self-punishment, anti-dissociation, suicide prevention, interpersonal influence, sensation-seeking, and interpersonal boundaries [12]. Building on this work, Cloutier and Nixon identified four functional categories of NSSI: internal emotion regulation, social influence, external emotion regulation, and sensation seeking. This classification was based on a comprehensive literature review, clinical expertise, and data from adolescent psychiatric inpatients. These categories were further validated in college student populations and clinical samples [13,14]. Among these functions, the emotional regulation function has received the most empirical support across clinical and non-clinical samples [12]. Additionally, higher functional scores of NSSI were associated with higher frequency of NSSI [13]. The function of NSSI reflects the purpose or effect achieved by the individual through self-injury, which provides insight into the motivations driving the behavior. Based on this, this study posits the following hypothesis:

**H1.**  *The frequency of NSSI is positively associated with the functions of NSSI.*

### 1.2. Self-Control and the Frequency of NSSI

Adolescent NSSI behavior seems to violate the innate motivation of human self-protection and deviate from the long-term goal of humans pursuing health and longevity [6]. Repeated NSSI is considered an addictive behavior [15]. Individuals who engage in NSSI often develop a certain level of dependence on this behavior and struggle to control it. Self-control is the ability of individuals to overcome or suppress their inner desires and adjust their behavior and thinking in order to achieve long-term goals [16,17]. Self-control has a wide range of effects on behavior, including behavioral school and work performance, eating and weight-related behaviors, addictive behaviors, interpersonal functioning, emotional regulation, well-being, and adaptation [18]. Individuals with higher scores on self-control show fewer impulse control problems and fewer reports of psychopathology [19]. Studies have found that self-control is a protective factor for suicidal thoughts and attempts [20], and previous studies have fully demonstrated the high correlation between suicide attempts and NSSI [2,7]. Studies on the relationship between self-control and NSSI are still limited, especially in clinical samples. High impulsivity and difficulty regulating emotions predicted more recent and frequent NSSI behavior in individuals [21,22]. Inadequate self-control of negative emotions and cognition may be associated with the frequent occurrence of NSSI. Therefore, a second hypothesis is proposed in this study:

**H2.**  *Self-control is negatively associated with the frequency of NSSI.*

### 1.3. The Mediating Role of the Function of NSSI

Integrated model theory proposed that NSSI is maintained as a way to quickly regulate negative emotions or influence others [23]. A growing body of research indicates that NSSI primarily serves to decrease adverse emotional and cognitive states [6]. The occurrence of NSSI is often preceded by an increase in negative emotions such as depression, anxiety, and loneliness [24]. Both emotion regulation and impulse control as important components of self-control have been found to play important roles in NSSI behaviors [21,25]. In this study, self-control is seen as the ability of an individual to suppress unwanted behaviors, and in the dual systems perspective, self-control is defined as a product of the reflective system, involving higher cognitive processes such as assessment, planning, and inhibition, and the ability to work against the impulsive system [26]. Individuals with less self-control may have more difficulty regulating negative emotions, and individuals with higher impulsivity may be more likely to adopt NSSI behaviors as coping strategies in the face of stress [1], which aligns with the purpose of NSSI. Consequently, self-control is not only associated with the frequency of NSSI but may also be linked to its function. Based on this, we propose hypothesis 3:

**H3.**  *NSSI function mediates the relationship between self-control and NSSI frequency.*

The majority of research on NSSI among adolescents has been derived from the general population, and there was high heterogeneity between studies [5]. Meanwhile, studies within psychiatric clinic settings typically concentrate on clinical risk factors and mechanism assessments [27,28]. The emergence of NSSI is not attributable to a single factor in isolation but is the intricate outcome of an array of interrelated and reciprocal protective and risk factors [2,25]. To our knowledge, the protective factors associated with this high-risk behavior have not been thoroughly investigated. A profound exploration of the protective factors associated with NSSI is essential for the crafting of efficacious prevention initiatives and intervention programs aimed at reducing NSSI behaviors among adolescents [29].

The objectives of this study were threefold. Firstly, it sought to delineate the characteristics of NSSI, including the frequency, severity, location, and function of NSSI, within a clinical adolescent population. Secondly, this study endeavored to examine the demographic and protective factors that distinguish adolescents engaging in NSSI from those who do not, within the context of outpatient facilities. Third, this study aimed to explore the relationship between self-control, NSSI function, and NSSI frequency, as well as to provide insights that could inform targeted interventions.

## 2. Materials and Methods

A total of 206 participants were recruited from outpatient adolescents receiving care at the psychiatric department of a provincial-level Class III Grade A hospital in China. This hospital represents the highest tier of medical institutions in the country, ensuring access to specialized psychiatric services. Recruitment was conducted randomly from adolescents attending routine outpatient consultations, including both new and returning patients, to ensure that the sample was representative. The study received approval from the responsible ethical committee.

Inclusion Criteria

Participants aged 12 to 18 years who were psychiatric outpatients and capable of comprehending the content of the questionnaires and independently completing the study were included. Participants and their families voluntarily agreed to participate in the study and provided informed consent by signing the consent form.

Exclusion Criteria

Participants who lacked the ability to understand the study materials or communicate effectively were excluded. Individuals with severe mental illnesses requiring inpatient treatment, as well as those who did not provide signed informed consent, were not eligible for the study.

### 2.1. Procedure

Recruitment took place between August and September 2024, with participants randomly selected from adolescents attending routine outpatient consultations at the hospital. An experienced chief psychiatrist recommended individuals who met the inclusion criteria to participate in the study. Three psychology graduate students then conducted face-to-face interviews and psychological assessments of the participants and collected data. Before participation, potential participants and their families were informed about the study’s purpose, procedures, potential risks and benefits, and their rights, including the option to withdraw at any time. To ensure full disclosure and voluntary participation, each participant and their relatives signed informed consent forms, confirming their understanding of the study details and willingness to participate. Participants then completed standardized questionnaires in a quiet, private setting, with a professional present to provide assistance if needed.

### 2.2. Measures

Sociodemographic data: Sociodemographic data were collected using a self-reported questionnaire, which included age, gender, residence, single-child status, monthly household income, and family structure.

Ottawa Self-Injury Inventory (OSI): This is a questionnaire developed by researchers at the University of Ottawa to assess the frequency, motivation, pattern, and related psychological factors of self-injurious behavior in adolescents. The frequency of NSSI was assessed with questions such as “How often in the past 6 months have you injured yourself, without the intention to kill yourself?”. Response options ranged from 1 (never) to 5 (daily). The NSSI function subscale contains 21 items in 4 dimensions, and each item is rated on a 5-point scale (ranging from 1, never, to 5, always). The dimensions are internal emotion regulation (e.g., to relieve feelings of sadness), social influence (e.g., to gain care and attention from others), external emotion regulation (e.g., to release anger), and sensation seeking (e.g., to experience excitement). Additionally, the OSI includes items that inquire about the specific types of self-injury (e.g., cutting, burning), the location of engaging in such behaviors (e.g., arm, hand), and the age of the first NSSI. This scale has been found to be reliable and valid in both clinical and undergraduate samples [13,14]. For the current sample, the Cronbach’s alpha ranged from 0.69 to 0.92.

The Self-Control Scale (SCS): This is a scale developed by the psychologist Tangney in 2004 to assess the self-control ability of individuals in daily lives [19]. The Self-Control Scale (SCS) consists of 36 items, divided into five dimensions. Sample items include “I’m easily tempted” and “I often can’t control my emotions”. Responses are rated on a 5-point Likert scale, ranging from 1 (completely inconsistent) to 5 (completely consistent). In this study, the Cronbach’s alpha was 0.94 for the total scale. Cronbach’s alpha coefficients for the sub-dimensions of the scale were found as follows: impulse control (α = 0.77), emotional regulation (α = 0.86), healthy habits (α = 0.86), distraction management (α = 0.79), and overall self-discipline (α = 0.79).

### 2.3. Data Analysis

Data analyses were conducted using SPSS 27.0, PROCESS 4.2, and AMOS 28.0. For analysis, participants were divided into two groups: the NSSI group and the non-NSSI group, based on whether they had ever engaged in NSSI. Pearson’s chi-square test, independent sample *t*-test, and one-way analysis of variance (ANOVA) were used to examine differences in sociodemographic characteristics and key variables between individuals who engaged in NSSI and those who did not. Additionally, the NSSI group was described in terms of frequency, location, method, and function of self-injury.

In order to explore the relationship between variables and the potential mechanisms, a mediation analysis was conducted in the NSSI group [30]. A structural equation model was constructed to test the relationships between predictors (self-control), potential mediators (the function of NSSI), and the dependent variable (the frequency of NSSI). Self-control was modeled as a latent variable comprising five observed indicators: impulse control, emotion regulation, distraction management, healthy habits, and overall self-discipline. The function of NSSI was modeled as a latent variable comprising four observed indicators: internal emotion regulation, social influence, external emotion regulation, and sensation seeking. The frequency of NSSI was included as an observed variable.

The reference criteria for good model fit were as follows: χ^2^/df < 3.0, comparative fit index (CFI) > 0.90, and root mean square error of approximation (RMSEA) < 0.10 [31]. The bootstrapping methodology proposed by Shrout and Bolger (2002) was applied, with 5000 bootstrap replications [32].

## 3. Results

### 3.1. Participant Characteristics

In this study, a total of 206 psychiatric outpatients were evaluated, the majority of whom were female (*n* = 135, 65.5%) and did not identify as an only child (*n* = 124, 60.2%). A significant proportion resided in urban areas (*n* = 120, 58.3%) and came from nuclear families (*n* = 136, 66%). The average age of the participants was 15.8 years (SD = 1.56).

Table 1 presents a detailed analysis of the sociodemographic and psychological characteristics distinguishing the NSSI group from the non-NSSI group (patients without NSSI). Individuals with a history of any form of NSSI behavior were included in the NSSI group, and individuals with no NSSI behavior at all were included in the non-NSSI group. A substantial majority of the adolescents in our sample, 77.18% (*n* = 159), reported a history of engaging in NSSI. Furthermore, 81.6% (*n* = 168) endorsed having suicidal thoughts, and 47.1% (*n* = 97) admitted to suicide attempts. Suicidal ideation (χ^2^ = 38.333, df = 1, *p* < 0.001) and suicide attempts (χ^2^ = 22.421, df = 1, *p* < 0.001) in the NSSI group were significantly higher than those in the non-NSSI group. Statistically significant differences were observed between the NSSI and non-NSSI groups in terms of gender (χ^2^ = 19.059, df = 1, *p* < 0.001), with females being over-represented in the NSSI group. In terms of monthly household income, there were significant differences in the distribution of monthly income between the two groups (χ^2^ = 45.886, df = 3, *p* < 0.001), with a higher proportion of participants in the NSSI group having a lower monthly income and a higher proportion of participants in the non-NSSI group having a higher monthly income.

Regarding age, the number of children in the family, family structure, and family residence, no significant differences were found between the groups. The self-control score of the NSSI group was significantly lower than that of the non-NSSI group (F = 27.458, df = 205, *p* < 0.001), suggesting that this psychological factor may play a pivotal role in the development and maintenance of NSSI behaviors.

Table 2 displays the frequencies, percentages, means, and standard deviations for the location, method, and function of NSSI within the NSSI group. The average age of onset for NSSI was 13.13 (SD = 2.25). In this sample, the body parts most commonly injured by adolescents were the arms (*n* = 127, 81.4%) and hands (*n* = 82, 52.6%). The most frequently used methods for NSSI were cutting (*n* = 96, 61.5%), scratching (*n* = 85, 54.5%), and beating oneself (*n* = 84, 53.8%). The mean score of NSSI function was 2.51, with a standard deviation of 0.70, indicating that NSSI function was at a moderate level in the sample.

### 3.2. Associations Among Frequency, Functions of NSSI, and Self-Control

Table 3 presents the correlation and descriptive data of each variable after controlling for sociodemographic parameters. NSSI frequency was positively correlated with the internal emotional regulation function, social influence function, external emotional regulation function, and sensation seeking function. The correlation analysis results support hypothesis 1 (H1): the frequency of NSSI is positively associated with the functions of NSSI. Self-control was negatively associated with NSSI frequency, internal emotional regulation function, social influence function, and external emotional regulation function. These results also support hypothesis 2 (H2): self-control is negatively associated with the frequency of NSSI. The pattern of correlation between variables is consistent with the pattern of variables relationship and is suitable for structural equation modeling analysis.

In order to obtain a continuous variable of the overall NSSI frequency, we used the weighted average method, which is a method in statistics that takes into account the relative importance or weight of each value in a data set when calculating an average. This method is particularly suitable for data collected at different time points or under different conditions. This study investigated the frequency of NSSI of participants in the past 1 month, the past 6 months, and the past 1 year, and it was reasonable to use the weighted average method to calculate the total NSSI frequency. Cronbach’s alpha of the NSSI frequency scale after the weighted average treatment was 0.892. The weighted average formula used to calculate the overall NSSI frequency is as follows:

Overall NSSI frequency = (Frequency in the past month × 0.5) + (Frequency in the past 6 month × 0.3) + (Frequency in the past year × 0.2)

### 3.3. Mediation Analysis

**Figure 1:**
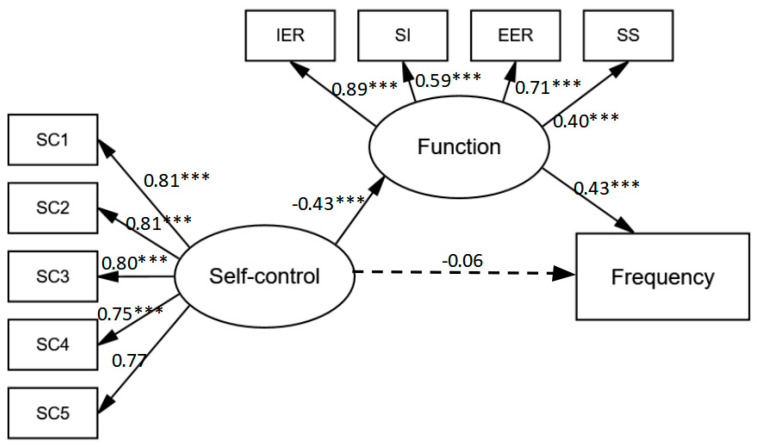
Mediating effects of functions in the relationship between self-control and the frequency of NSSI. (IER = internal emotion regulation, SI = social influence, EER = external emotion regulation, SS = sensation seeking, SC1 = impulse control, SC2 = emotional regulation, SC3 = healthy habits, SC4 = distraction management, and SC5 = overall self-discipline. *** *p* < 0.001.)

A structural equation model was used to investigate the relationship between the NSSI function, frequency, and self-control of adolescents (Figure 1). The fit of the measurement model is as follows: CMIN/DF = 2.493, CFI = 0.925, and RMSEA = 0.098. The measurement model was a mediocre fit.

Mediation analysis showed that there was a significant total effect and indirect effect between self-control, function, and the frequency of NSSI (Table 4). Moreover, the direct effect between self-control and the frequency of NSSI was not significant. Therefore, we hold the opinion that the function of NSSI completely mediates the effects of self-control on the frequency of NSSI, accounting for 62.07% of the variance in the mediation analysis, which supports our hypothesis 3 (H3): NSSI function mediates the relationship between self-control and NSSI frequency.

## 4. Discussion

NSSI in adolescents, defined as deliberate self-harm without suicidal intent, is a multifaceted phenomenon influenced by psychological, social, and biological factors. Extensive research has explored these contributing factors, underscoring the complexity of this behavior. In summary, this study achieved three primary objectives. First, it provided a detailed characterization of NSSI in a clinical adolescent population, examining its frequency, severity, location, and function. Second, it identified demographic and protective factors distinguishing adolescents who engage in NSSI from those who do not within outpatient settings. Finally, this study explored the relationship between self-control and NSSI, in particular, the function of NSSI mediated the relationship between self-control and NSSI frequency. These findings contribute to a nuanced understanding of NSSI in adolescents, highlighting key factors that may guide targeted interventions and support in clinical contexts.

The results indicate that the NSSI is quite prevalent, as it has a detection rate of 77.18% in the psychiatric outpatient population, which deserves our attention as this rate is far higher than the clinical detection rate of 40–60% mentioned above [6]. A retrospective study conducted in the same region as this study reported a 43.27% detection rate in 2022, suggesting a rising trend in NSSI among adolescents [33]. Methodological factors may explain much of this variability; for example, Swannell et al. (2014) found that 51.6% of the heterogeneity in prevalence estimates could be attributed to differences in methodology [5]. Nock (2010) similarly noted that broader definitions of NSSI, the use of self-report scales instead of interviews, and sampling from specific populations tend to yield higher prevalence rates [6]. The detection criteria for NSSI in this study are based on the frequency of NSSI in the Ottawa Self-Injury Inventory, which are more relaxed than those in previous studies. Additionally, the incidence of NSSI may have increased in recent years, coinciding with the rising global prevalence of mental health problems among children and adolescents during the COVID-19 pandemic [34]. The gender difference is consistent with previous studies, that is, women have a higher detection rate of NSSI than men [35,36].

Previous studies have found that NSSI function is significantly correlated with NSSI frequency except for the external emotion regulation factor [34]. In this study, all four factors of NSSI function were significantly correlated with NSSI frequency, which further supports the four-factor model of NSSI function and the application of the OSI NSSI function scale in clinical samples. Among the four sub-dimensions of NSSI function, the scores of internal and external emotion regulation were higher, suggesting that individuals may use NSSI more to regulate and cope with internal and external emotional distress. This finding is consistent with the work of Klonsky (2007), who pointed to emotion regulation as a major function of NSSI [12]. NSSI may be favored by individuals who struggle with emotional regulation because it is an action that meets certain psychological needs, compared to other behaviors that require time, materials, and money (for example, drinking or using drugs). Once individuals begin to implement NSSI, some may begin to see it as an effective coping mechanism or even as a reliable way to achieve emotional regulation [11,22,23].

Expanding on the concept of NSSI, it can be seen as a maladaptive coping mechanism that adolescents may employ to regulate overwhelming emotions or to communicate distress in the absence of more effective strategies [15]. This behavior, while harmful, serves a functional purpose for the individual in the short term, such as providing a sense of control or relief from emotional pain. However, it is detrimental to the individual’s long-term health and well-being, as it can lead to chronic physical injuries, mental health issues, and social isolation. People struggle between long-term goal-inhibiting behaviors and immediate impulses to seek pleasure and satisfaction. Individuals with lower levels of self-control are more likely to abandon long-term goals to achieve short-term pursuits [19].

Impulsivity is commonly regarded as an indicator of low self-control [17,24]. However, recent research found that adolescents engaging in NSSI exhibited enhanced reactive inhibition [37], challenging the traditional view that NSSI is purely an impulsive behavior. This finding highlights the paradox of NSSI—behaviors that contradict the human instinct for self-preservation—while emphasizing the need to reconsider the role of self-control in NSSI. Although previous studies have found a significant correlation between emotional regulation and impulse control and NSSI [22,26,27,38], we still need to be cautious about the impact of self-control on NSSI. In fact, the emergence of NSSI is not attributable to a single factor in isolation but is the intricate outcome of an array of interrelated and reciprocal protective and risk factors [2,24]. According to the dual-system model of self-control, behavior is governed by the interaction between the impulsive system and the reflective system [24]. The impulsive system is responsible for generating impulsive behavior, accompanied by strong motivation. When individuals perform NSSI, they are often accompanied by a strong motivation to relieve negative emotions [12,13], which our research also showed. The reflective system is a process in which individuals make deliberate judgments and evaluations, formulate strategic action plans for goal pursuit, and suppress or override preset responses. Future research should explore whether the protective effect of self-control on NSSI lies in reducing NSSI impulses or in facilitating timely adjustments and adaptive responses to such behaviors.

Previous studies have proposed that empirical studies on self-control mainly focus on diet health and personal achievement [17,20], and there are very few studies in other fields, such as self-injury behaviors. This study discusses the role of self-control in NSSI, and the mediation analysis found that the relationship between self-control and the frequency of NSSI was fully mediated by the function of NSSI. The self-control defined in this paper is trait self-control, which refers to the stable and inter-individual differences in self-control ability and is a stable personality trait [39]. This suggests that trait self-control may be associated with the motivation behind NSSI, potentially acting as a protective factor, which could inform intervention strategies. Self-control can be cultivated through regular exercise, just like shaping a person’s character, and can be improved even in adulthood [40]. Thus, fostering self-control can be an effective way to prevent NSSI among adolescents if the psychological dimension is taken into account when formulating national policies.

There are several limitations to this study. First, as a cross-sectional study, it cannot establish causal relationships and is susceptible to confounding variables. Consequently, this study can only infer the correlation between self-control, NSSI function, and NSSI frequency, rather than determine causal pathways. Additionally, there may be uncontrolled variables that influence the relationships among self-control, NSSI function, and NSSI frequency. Future research should explore the causal relationships between these variables using longitudinal or randomized controlled designs. Second, while this study focused on the protective role of self-control, it did not account for a broader range of control variables. As previously discussed, NSSI is a multifaceted behavior influenced by a complex interplay of protective and risk factors. To provide a more comprehensive understanding of NSSI, future research should include a wider array of both extreme risk and protective factors. Another limitation is the reliance on self-reported measures to assess participants’ NSSI behaviors, self-control, and clinical variables. Despite rigorous procedures, self-report methods are inherently prone to bias. Individuals who engage in NSSI may not fully understand or may find it difficult to accurately express the underlying reasons for their actions, leading to potential inaccuracies in self-assessments. Additionally, some participants may provide socially desirable responses or conceal their true motivations due to embarrassment [15]. Moreover, the static nature of cross-sectional data does not allow for the observation of changes over time, which is essential for understanding the development and progression of complex behaviors such as NSSI. Future studies could consider using dynamic methods, such as ecological momentary assessment (EMA), to capture real-time fluctuations in NSSI behavior. Task-based measurement methods, which have been shown to be more reliable than self-reports, could also enhance data validity [37]. Finally, this study distinguished only between NSSI and non-NSSI groups, a relatively simplistic classification that may be influenced by recall bias in participants’ self-reports of their behaviors. Future research should further investigate the characteristics and protective factors of individuals with varying degrees of NSSI severity (e.g., mild vs. severe), frequency (e.g., occasional vs. repeated), and stage (e.g., current vs. past). This would help determine whether self-control protects individuals from more severe or repeated NSSI and whether it serves as a key factor in motivating individuals to stop engaging in NSSI.

## 5. Conclusions

This study revealed a high prevalence of NSSI among adolescents in psychiatric outpatient care, with significant differences in gender, family income, and self-control levels between the NSSI and non-NSSI groups. Notably, self-control was found to be significantly associated with NSSI behavior, and this relationship was fully mediated by the function of NSSI. Specifically, lower self-control was linked to more frequent NSSI behaviors, mediated entirely by the functional role of NSSI. Conversely, higher self-control was associated with a lower likelihood of NSSI and better emotional regulation, suggesting a potential protective effect of self-control against NSSI.

These findings underscore the importance of addressing both self-control and the function of NSSI in developing prevention and intervention strategies. They also provide a theoretical foundation for designing targeted psychological interventions to reduce NSSI behaviors in adolescents.

## Figures and Tables

**Table 1:** Demographic characteristics of adolescent outpatients.

	Total (*n* = 206)		NSSI (*n* = 159)		Non-NSSI (*n* = 47)	
	*N*	%	*N*	%	*N*	%
Female	135	65.5	117	73.6	18	38.3
Single-child	82	39.8	62	39.0	20	42.6
Urban	120	58.3	91	57.2	29	61.7
Monthly income ($)						
685-	62	30.1	51	32.1	11	23.4
685–1370	80	38.8	55	34.6	25	53.2
1370–2741	44	21.4	3	1.9	2	4.3
2741-	15	7.3	27	17.0	6	12.8
Family structure						
Nuclear	136	66.0	104	65.4	32	68.1
Backbone	44	21.4	30	18.9	14	29.8
Joint family	1	0.5	1	0.6	0	
Single-parent	16	7.8	13	8.2	3	6.4
Reorganized	8	3.9	7	4.4	1	2.1
Suicidal Thought	168	81.6	142	89.3	26	55.3
Suicidal Attempt	97	47.1	88	55.3	9	19.1
	*M*	*SD*	*M*	*SD*	*M*	*SD*
Age	15.08	1.56	15.01	1.55	15.30	1.60
Self-control	2.62	0.60	2.50	0.54	2.98	0.63

**Table 2:** Descriptive statistics for the NSSI group (*n* = 159).

	*N*	%		*N*	%
Location			Method		
arm	127	81.4	Cutting	96	61.5
hand	82	52.6	Scratching	85	54.5
face	41	26.3	Beating	84	53.8
lip	27	17.3	Biting	71	45.5
	*M*	*SD*	Piercing of body parts	33	21.2
Function	2.51	0.70	Burning	4	2.6
Internal ER	2.96	0.93	Headbanging	58	37.2
Social influence	1.99	0.80	Hair pulling	37	23.7
External ER	3.31	0.97	Severe nail biting	40	25.6
Sensation seeking	2.02	1.03	Hindering healing	50	32.1
Self-control	2.45	1.08			

**Table 3:** Correlation coefficients of study variables.

	1	2	3	4	5	6
1. NSSI frequency	1					
2. Self-control	−0.234 **	1				
3. Internal ER	0.39 **	−0.346 **	1			
4. Social Influence	0.318 **	−0.264 **	0.526 **	1		
5. External ER	0.31 **	−0.348 **	0.656 **	0.389 **	1	
6. Sensation Seeking	0.267 **	−0.156	0.369 **	0.327 **	0.182 *	1

* *p* < 0.05, ** *p* < 0.01.

**Table 4:** Bootstrap test results.

Path	Effect	SE	LLCI	ULCI	Meditated Percentage
Total effect	−0.4627 **	0.1566	−0.7721	−0.1533	
Direct effect	−0.1755	0.1559	0.2620	−0.4834	
Indirect effect	−0.2872 **	0.8070	−0.4553	−0.1410	62.07%

** *p* < 0.05.

## Data Availability

The data presented in this study are available on request from the corresponding author. The data are not publicly available due to privacy.
